# Etiology of Genital Ulcer Disease in a Sexually Transmitted Infection Reference Center in Manaus, Brazilian Amazon

**DOI:** 10.1371/journal.pone.0063953

**Published:** 2013-05-21

**Authors:** Felipe Gomes Naveca, Meritxell Sabidó, Tatiana Amaral Pires de Almeida, Elaine Araújo Veras, Matilde del Carmen Contreras Mejía, Enrique Galban, Adele Schwartz Benzaken

**Affiliations:** 1 Fundação Alfredo da Matta, Manaus, Brazil; 2 Instituto Leônidas e Maria Deane – Fiocruz Amazônia, Manaus, Brazil; 3 Fundação de Medicina Tropical Doutor Heitor Vieira Dourado (FMT-HVD), Manaus, Brazil; 4 Fundació Sida i Societat, Barcelona, Spain; 5 Universidade Federal do Amazonas, Manaus, Brazil; 6 Facultad de Medicina Calixto García, La Habana, Cuba; 7 UNAIDS, Brasilia, Brazil; University of Alabama at Birmingham, United States of America

## Abstract

**Objectives:**

To determine the etiology and factors associated with genital ulcer disease (GUD) among patients presenting to a sexually transmitted infections clinic in Manaus, Brazil; and to compare a multiplex polymerase chain reaction (M-PCR) assay for the diagnosis of GUD with standard methods.

**Methods:**

Ulcer swabs were collected and used for Tzanck test and processed in an M-PCR to detect herpes simplex virus (HSV-1/2), *Treponema pallidum (T. pallidum)*, and *Haemophilus ducreyi (H. ducreyi)*. Sera were tested for HIV and syphilis antibodies. Multivariable analysis was used to measure the association between clinical aspects and GUD. M-PCR results were compared with syphilis serology and Tzanck tests.

**Results:**

Overall, 434 GUD samples were evaluated, 84.8% from men. DNA from HSV-2 was detected in 55.3% of GUD samples, *T. pallidum* in 8.3%, HSV-1 in 3.2%, and 32.5% of GUD specimens were negative for the DNA of all three pathogens. No cases of *H. ducreyi* were identified. HIV serology among GUD patients was 3.2%. Treponemal antibodies and Tzanck test positivity for genital herpes was detected in 25 (5.8%) and in 125 (30.3%) of GUD patients, respectively. In multivariable analysis genital herpes etiology by M-PCR was associated with the vesicular, multiple and recurrent lesions whereas *T. pallidum* with non-vesicular, non-recurrent lesions. Compared to M-PCR, syphilis serology was 27.8% sensitive and 96.2% specific whereas Tzanck test was 43.8% sensitive and 88.9% specific.

**Conclusions:**

The predominance of genital herpes etiology suggests a revision of existing national syndromic treatment guidelines in Brazil to include antiherpetic treatment for all GUD patients. The use of M-PCR can significantly improve the diagnosis of GUD and provide a greater sensitivity than standard diagnostics.

## Introduction

The three pathogens most frequently associated with genital ulcer disease (GUD) are herpes simplex virus type 2 (HSV-2), *Treponema pallidum*, and *Haemophilus ducreyi*. [1], [2], [3] Brazil is currently employing adapted World Health Organization (WHO) syndromic management guidelines, [4] an approach based primarily on the clinical presentation of ulceration. GUD algorithm includes antiviral treatment for genital herpes for anyone with typical symptoms/signs and suggests that all GUD patients receive treatment for HSV-2 in settings where genital herpes is responsible for 30% or more of GUD cases. In the absence of vesicular lesions, GUD are treated syndromically for both syphilis and chancroid.

However, the etiologic pathogens of GUD vary temporally and geographically and current syndromic criteria may not reflect changes in the prevalence of specific pathogens. In Brazil, epidemiological data on sexually transmitted infections are scarce. In a study in a primary health care centre in North East Brazil, GUD accounted for 4.8% of cases of genital syndromes. [5] In a multicenter study conducted in 6 Brazilian capitals, the prevalence of syphilis was 2.6% among pregnant women and 3.4% among patients attending STI clinics. [6] GUD from syphilis and HSV-2 are associated with HIV acquisition and transmission. [7].

Clinical diagnosis is an unreliable means of distinguishing between different GUD due to atypical presentation, the occurrence of mixed infections and HIV infection. [8], [9] Gold-standard laboratory tests for these organisms are only performed in a few laboratories. [10], [11], [12] For example, in Brazil, only the Venereal Disease Research Laboratory (VDRL) test for syphilis detection is routinely and widely available. Thus, the diagnosis of GUD is undertaken in the context of syndromic management, an approach based primarily on the clinical presentation of ulceration. [13] Therefore, accurate laboratory tests are needed for the selection of appropriate treatment strategies.

Use of nucleic acid amplification tests has facilitated the detection of several etiologic agents that were uncultivable. PCR can provide a more sensitive microbial diagnosis, with direct detection of the pathogen from the clinical sample. [14], [15] Multiplex PCR (M-PCR) assay makes best use of limited amounts of clinical material since it allows the simultaneous amplification of DNA from three pathogens from a single swab specimen of genital ulcer secretions. [14] M-PCR has shown a higher sensitivity than standard diagnostic tests for the detection of HSV, *H. Ducreyi* and *T. pallidum* from genital ulcers.

The aim of the study was to determine the etiologic cause of genital ulcers in an STI clinic in Manaus, Brazilian Amazon, in order to provide necessary information for ensuring that the syndromic guidelines are in line with the current disease patterns. We introduced M-PCR diagnostic method and compared it to standard methods that have been previously used in this setting.

## Methods

### Study Setting and Participants

The study was conducted at the Fundação Alfredo da Matta (FUAM), which runs a reference outpatient clinic, specialized in STI care in Manaus, Brazil, the largest city in the Amazon Region. Consecutive, non-selected patients with clinical symptoms of GUD presenting at FUAM, as evidenced by disruption of genital mucous membranes or epithelium were invited to participate in the study between May 2008 and September 2009. Patients with previous or ongoing antibiotic therapy and pregnant women were included. The study protocol was approved by the Research Ethics Committee of FUAM.

### Data and Specimens Collection and Preparation

The attending physician explained the study and obtained written informed consent. The physicians had undergone special training in STIs and their syndromic management. Participation included the collection of sociodemographic (age, sex) and clinical data (time from the onset, recurrence) using a standardized form, followed by physical examination (number and appearance of the lesions) and sample collection. Among women, vulvar, vaginal and cervical examination was also conducted. All treatment was dispensed according to the national syndromic management guidelines. [16] Patients were asked to return eight days later.

The ulcers were cleaned with saline and a swab from the base of each lesion was collected and smeared on a slide for cytodiagnosis of herpetic infection (Tzanck test). A second swab was immediately placed in a microtube with 4M guanidium thyocyanate (Invitrogen, Carlsbad, CA, USA) and processed for DNA isolation by the phenol/chloroform/isopropanolol method. [17] Each DNA pellet was resuspended in 200 µL of TE buffer (10 mM Tris-HCI, pH 8.0, 0.1 mM EDTA). In addition, blood was obtained for both syphilis and HIV serologies.

### Multiplex *T. pallidum, H. ducrey* and HSV Polymerase Chain Reaction (M-PCR)

Total DNA was extracted and subsequently stored at −20°C until we performed M-PCR based on previously described protocol [14] but with a major adaptation. Neither biotinylated primers nor target-specific oligonucleotides probes were used. Instead a specially designed DNA polymerase for higher sensitivity and specificity on M-PCR applications (AccuPrime – Invitrogen) was used in a conventional PCR format combined with a restriction endonuclease digestion step with *Hae*III, according to manufacturer’s recommendations (Invitrogen). This step was *in silico* designed with the aid of the software REviewer™ (freely available at the website http://www.fermentas.com/reviewer/app) and included in order to discriminate between amplicons of HSV-1, HSV-2 or HD because they have equal or very close sizes: 432bp for HSV-1 or HSV-2 and 437bp for HD. After digestion, fragments of 104 and 183bp were expected in HSV-1 cases. 104 and 275bp for HSV-2, and 155 plus 205bp in HD cases.

All PCR reactions were performed in a final volume of 25 µL, containing 2.5 µL of 10X AccuPrime buffer II, 0.3 µM of each primer, 1.5 mM of MgCl_2_, 2.5 U of AccuPrime DNA polymerase, ultra-pure distillated water to a final volume of 20 µL and five microliters of each ressuspended DNA. PCR reactions were conducted in an Eppendorf thermocycler (Eppendorf Mastercycler, Hamburg, Germany). The PCR program consisted of initial denaturation for 2 minutes followed by 40 cycles of denaturation at 94°C, annealing at 60°C and polymerase extension at 72°C (each step lasting 30 seconds), and a final extension of seven minutes at 72°C. The reaction was kept at 4°C until analysis. No-template controls were included on each lot of the specimens tested.

Ten microliters of M-PCR amplicons and 0.5 µL of a 100bp DNA ladder (Invitrogen) were electrophoresed in 1% agarose gels stained with SYBR Safe DNA gel stain (Invitrogen) according to the manufacturer’s recommendations and visualized with a blue-light transilluminator (Safe Imager - Invitrogen). Electrophoresis data were recorded with a digital camera.

Two sizes of amplicons were expected: a 220bp DNA fragment corresponding to the amplification of TP or another near 430bp corresponding to HSV-1/HSV-2 or HD. In order to discriminate between HSV-1, HSV-2 or HD specific-products, all samples that showed an amplicon around 430bp were submitted to the restriction enzyme digestion protocol with *Hae*III designed in this study. Five microliters of each amplicon were mixed with 10 units of *Hae*III (Invitrogen) and then incubated at 37°C for 2 hours followed by a step of 80°C during 20 minutes for enzyme inactivation. Digested amplicons were electrophoresed in 2% agarose gels as described for M-PCR products.

As for the endogenous control for PCR, samples that were negative in the M-PCR were submitted to a singleplex PCR reaction for a human housekeeping gene (beta-actin). Reaction was set up with Platinum PCR Master Mix according to the manufacturer’s instructions (Invitrogen). PCR primers and cycling conditions were used as described elsewhere. [18] The visualization of a single DNA fragment of 431bp ruled out a false-negative result due to PCR inhibition.

Specimens with discordant duplicates were reamplified and reanalyzed until the replicates were concordant.

### Comparative Diagnostics

Serum samples underwent syphilis testing initially with VDRL (Venereal Disease Research Laboratory, Wiener Laboratorios, Rosario, Argentina) followed by FTA-Abs (Fluorescent Treponemal Antibody Absorbed Test, WAMA Diagnóstica, São Carlos, SP, Brazil) for those with a positive VDRL. Syphilis infection was defined as sera reactive with both VDRL and FTA-Abs. HIV-1 and HIV-2 serum antibodies were detected by GENSCREEN HIV 1/2 v.2 ELISA (BIORAD, Rio de Janeiro, Brazil). Positive samples were confirmed by a second ELISA (GENSCREEN PLUS Ag/Ab, BIORAD, Rio de Janeiro, Brazil) and an indirect anti-HIV-1 immunofluorescent assay (Biomanguinhos, Rio de Janeiro, RJ, Brazil). Discrepant results were resolved by Western Blot (GLD HTLV BLOT 2.4, Genelabs Diagnostics, Singapore).

The Tzanck smear was examined by light microscopy and herpetic infection was diagnosed when epithelial cells showed characteristic and typical herpetic changes. [19] Although it is known to perform poorly, the Tzanck test is used in daily practice at the FUAM clinic and is recommended by the Brazilian Ministry of Health for the diagnosis of herpes. [16].

### Statistical Analysis

Data were analysed using STATA version 10.0 (StataCorp, College Station, Texas, USA).

Comparisons between men and women were examined using Pearson Chi square for categorical variables, Fisher’s exact test for small numbers, and Wilcoxon rank-sum test for continuous variables. Risk factors for HSV (including HSV-1 and HSV-2) and *T. pallidum* were identified using multivariable analysis: all factors with a p-value <0.05 in the univariate analysis were included in a logistic regression model following a backward stepwise procedure to retain only factors with a likelihood ratio test p-value ≤0.05 in the final model.

To compare proportions of detectable swabs in the M-PCR assay versus syphilis serology and HSV positive samples by Tzanck smear, Pearson Chi square tests were used and the resulting p-value is reported. The performance characteristics (sensitivity, specificity, and predictive values) of the tests were calculated according to standard methods.

## Results

### Patients’ Characteristics

During 17 months, we included 434 patients presenting at the STI clinic with a clinical picture of GUD. All of them were analyzed by M-PCR and were included in computing the prevalence estimates. However, three patients did not give a blood sample and 21 lacked Tzanck tests; therefore they were excluded from comparative analysis.

The ratio of males to females was 5.5∶1 and the median age was 27 years (interquartile range: 22–35 years) (Table 1). Almost a quarter (22.7%) of patients reported a history of genital ulcers before the episode leading to consultation, 84.9% consulted within 14 days of the onset of the ulcer, 58.4% presented with multiple lesions, and 23.3% had vesicular lesions. Males were significantly older (p = <0.001) and more likely to present with multiple lesions (p = 0.05). Out of 405 patients accepting HIV testing, 13 (3.2%) were HIV seropositive, with the prevalence in males (3.5%) being higher than in females (1.6%).

**Table 1:** Characteristics of Study Population Attending an STI clinic in Manaus, Brazil, by sex.

Characteristics	Total (n = 434)	Male (n = 368)	Female (n = 66)	p Value*
	n (%)	n (%)	n (%)	
Median age (IQR), years (n = 432) †	27 (22–35)	27 (23–36)	23.5 (19–31)	**<0.001** ††
Had genital ulcers prior				
No	324 (77.3)	270 (76.3)	54 (83.1)	0.2
Yes	95 (22.7)	84 (23.7)	11 (16.9)	
No. of days sore before consultation				
≤14	366 (84.9)	306 (83.6)	60 (92.3)	0.07
>14	65 (15.1)	60 (16.4)	5 (7.7)	
No. of ulcers present				
1	179 (41.6)	159 (43.6)	20 (30.8)	**0.05**
>1	251 (58.4)	206 (56.4)	45 (69.2)	
Appearance				
Non vesicular	332 (76.7)	284 (77.4)	48 (72.7)	0.4
Vesicular	101 (23.3)	83 (22.6)	18 (27.3)	
HIV serostatus				
Negative	392 (96.8)	329 (96.5)	63 (98.4)	0.4
Positive	13 (3.2)	12 (3.5)	1 (1.6)	

IQR: interquartile range.

* Using Pearson test unless specified.

† 2 men had missing value in age.

†† Wilcoxon rank-sum test.

Bold values indicate statistical significance (p<0.05).

### Prevalence

Using M-PCR, one or more pathogens were detected in 67.5% of swab specimens collected from 434 patients with symptomatic genital ulcers (Table 2). Altogether, 55.3% of the samples were positive for HSV-2, 8.1% for *T. pallidum*, 3.2% for HSV-1, 0.5% for dual TP/HSV-2 and 0.2% for dual TP/HSV-1. The remaining 32.5% were undetectable for DNA of each of the four STI pathogens. Almost a third (27.3%) of patients with ulcers with no detectable pathogen reported having had ulcers in the past. At the time of presentation, most patients with HSV did not have vesicles (35.7% HSV-1 and 31.8% HSV-2). Thirteen (36.1%) of the GUDs caused by *T. pallidum* showed multiple lesions.

**Table 2:** Patients Demographics, Clinical Characteristics and Distribution of Genital Ulcer-Causing Pathogens by M-PCR.

Demographic or ClinicalCharacteristics	HSV-1	HSV-2	TP	HSV/TP	ND
	n (%)	n (%)	n (%)	n (%)	n (%)
Total (n = 434)	14 (3.2)	240 (55.3)	36 (8.3)	3 (0.7)	141 (32.5)
Gender					
Male (n = 368 )	8 (2.2)	197 (53.5)	35 (9.5)	3 (0.8)	125 (34.0)
Female (n = 66)	6 (9.1)	43 (65.2)	1 (1.5)	0 (0.0)	16 (24.2)
Age in years (n = 432)					
<25 (n = 176)	10 (5.7)	111 (63.1)	7 (3.9)	3 (1.7)	45 (25.6)
25–34 (n = 142)	3 (2.1)	72 (50.7)	16 (11.3)	0 (0.0)	51 (35.9)
≥35 (n = 114)	1 (0.9)	56 (49.1)	13 (11.4)	0 (0.0)	44 (38.6)
Had genital ulcers prior					
No (n = 324)	12 (3.7)	165 (50.9)	32 (9.9)	3 (0.9)	112 (34.6)
Yes (n = 95)	1 (1.1)	67 (70.5)	1 (1.1)	0 (0.0)	26 (27.3)
Duration of ulcers in days					
≤14 (n = 366)	13 (3.5)	214 (58.5)	30 (8.2)	3 (0.9)	106 (28.9)
>14 (n = 65)	1 (1.5)	23 (35.4)	6 (9.2)	0 (0.0)	35 (53.9)
Number of ulcers present					
1 (n = 179)	4 (2.2)	75 (41.9)	23 (12.9)	0 (0.0)	77 (43.0)
>1 (n = 251)	10 (4.0)	162 (64.5)	13 (5.2)	3 (1.2)	63 (25.1)
Appearance					
Non vesicular (n = 332)	9 (2.7)	163 (49.1)	35 (10.5)	2 (0.6)	122 (36.8)
Vesicular (n = 101)	5 (4.9)	76 (75.3)	1 (1.0)	0 (0.0)	19 (18.8)
HIV serostatus					
Negative (n = 392)	13 (3.3)	217 (55.4)	29 (7.4)	2 (0.5)	130 (33.2)
Positive (n = 13)	0 (0.0)	8 (61.5)	2 (15.4)	0 (0.0)	3 (23.1)

HSV-1: herpes simplex virus type 1; HSV-2: herpes simplex virus type 2; TP: *T. pallidum*; HSV: herpes simplex virus type 1 and type 2; ND: pathogen not detected by M-PCR.

Of the 405 patients who tested for HIV, the seropositivity was higher among GUD patients with pathogens detectable by M-PCR (2.5%) than among the patients with no microbiological diagnosis determined (0.7%); however this finding was not statistically significant. The prevalence of ulcers with no detectable etiology did not differ among HIV-infected and –uninfected patients.

### Risk Factors Associated with Herpes Simplex Virus and *T. pallidum*

Table 2 presents the distribution and Table 3 shows the crude and adjusted odds ratios for the association between demographic and clinical characteristics of participants with M-PCR bases diagnoses of herpes simplex virus (including HSV-1 and HSV-2) and *T. pallidum*, compared with those without these diagnoses. Patients diagnosed with HSV by M-PCR were significantly more likely to have had GUD in the past (aOR 2.16 95% CI 1.26−3.66, p = 0.005), to consult earlier (aOR 0.54, 95 CI% 0.30−0.98, p = 0.04), and to present multiple (aOR 1.90, 95% CI 1.22−2.95, p = 0.004) and vesicular lesions (aOR 2.66, IC 95% 1.50−4.7, p = 0.001). In our sample, compared to the reference group (<25 years), older age was significantly less likely to show clinical GUD (for age group 25–34 years: aOR 0.48, 95% CI 0.29−0.79, p = 0.004, and for age group≥35 years: aOR 0.53, 95% CI 0.321−0.90, p = 0.02).

**Table 3:** Tabla 3. Demographic and Clinical Characteristics Associated with Genital Ulcer Diseases by M-PCR Etiologic Diagnosis among 434 Patients attending to the STI Clinic in Manaus, Brazil.

Variable	HSV				TP			
	OR (95% CI)	p Value	AOR (95% CI)	p Value	OR (95% CI)	p Value	AOR (95% CI)	p Value
Gender								
Male	1		1		1		1	
Female	2.29 (1.26–4.15)	**0.005**	1.83 (0.97–3.46)	0.06	0.15 (0.02–1.10)	**0.03**	0.16 (0.02–1.24)	0.08
Age in years								
<25	1		1		1		1	
25–34	0.51 (0.32–0.81)	**0.004**	0.48 (0.29–0.79)	**<0.001**	3.07 (1.21–7.76)	**0.01**	3.21 (1.19–8.70)	**0.02**
≥35	0.45 (0.28–0.75)	**0.001**	0.53 (0.31–0.90)	**0.02**	3.11 (1.19–8.14)	**0.02**	3.24 (1.15–9.12)	**0.03**
Had genital ulcers prior								
No	1		1		1		1	
Yes	2.09 (1.27–3.46)	**0.003**	2.16 (1.26–3.66)	**<0.001**	0.10 (0.01–0.74)	**<0.001**	0.09 (0.01–0.69)	**0.02**
Duration of ulcers (days)								
≤14	1		1		1			
>14	0.36 (0.21–0.63)	**<0.001**	0.54 (0.30–0.98)	**0.04**	1.14 (0.45–2.86)	0.78	0.71 (0.26–1.90)	0.5
Number of ulcers present								
1	1		1		1			
>1	2.76 (1.83–4.15)	**<0.001**	1.90 (1.22–2.95)	**<0.001**	0.37 (0.18–0.76)	**<0.001**	0.56 (0.25–1.23)	0.15
Appearance								
Non vesicular	1		1		1		1	
Vesicular	3.77 (2.17–6.54)	**<0.001**	2.66 (1.50–4.73)	**0.001**	0.08 (0.01–0.64)	**<0.001**	0.10 (0.01–0.78)	**0.02**
HIV serostatus								
Negative	1				1			
Positive	0.96 (0.75–1.23)	0.75	1.73 (0.50–6.03)	0.4	1.58 (1.00–2.47)	0.05	1.73 (0.32–9.40)	0.5

OR: crude odds ratio; AOR: Adjusted odds ratio; CI: confidence interval.

Bold values indicate statistical significance (p<0.05).

With regards to risk factors for *T. pallidum*, younger age, not having had a GUD in the past and presenting non-vesicular lesions were significantly associated with a positive *T. pallidum* result after adjustment.

### Comparison between Syphilis Serology, Tzanck Test and M-PCR Results

Of the 434 specimens tested for syphilis, 25 (5.8%) were VDRL positive and confirmed by FTA-Abs (Table 4). Ten of these 25 samples (40.0%) were also *T. pallidum* positive by M-PCR. Of the remaining 15 samples (60.0%), syphilis serology positive M-PCR negative samples, HSV DNA was detected in 8 ulcer swabs. Syphilis serology and M-PCR results were significantly related (p<0.001).

**Table 4:** Comparison of M-PCR with syphilis serology and Tzanck test.

	Syphilis serology				Tzanck test			
	+(%)	−(%)	Total	p Value	+(%)	−(%)	Total	p Value
PCR +	10 (40.0)	26 (6.4)	36 (8.3)		106 (84.8)	136 (47.2)	242 (58.6)	
PCR -	15 (60.0)	383 (93.6)	398 (91.7)	**<0.001**	19 (15.2)	152 (52.8)	171 (41.4)	**<0.001**
Total	25 (100)	409 (100)	434 (100)		125 (100)	288 (100)	413 (100)	

Syphilis serology was considered positive if both the VDRL and the FTA-Abs were positive. Tzanck test was considered positive (i.e. diagnosis of herpetic infection) if epithelial cells showed characteristic and typical herpetic changes.

Bold values indicate statistical significance (p<0.05) using chi-square test.

HSV was diagnosed using the Tzanck test, with 30.3% (125/413) showing a positive result. In 106 (84.8%) of the ulcer swabs showing a Tzanck test result, HSV specific DNA was detected. Of the remaining 19 HSV Tzanck positive M-PCR negative samples, *T. pallidum* was detected for one ulcer swabs and a mixed infection TP/HSV-2 in another ulcer swab. Conversely, 136 out of 288 HSV Tzanck negative samples (47.2%) contained HSV-specific DNA as determined by M-PCR. Tzanck test results and M-PCR were significantly related (p<0.001).

Of the 141 patients with undetectable STI pathogens by PCR, 16 (11.3%) were Tzanck test positive, 6 (4.3%) had a positive syphilis serology, 1 (0.7%) showed dual VDRL/FTA-Abs and Tzanck test positivity and 118 (83.7%) had negative result in Tzanck test or in syphilis serology.

### Performance of the Syphilis Serology and Tzanck Test

The sensitivity of the syphilis serology compared with M-PCR was 27.8% (95% CI: 14.2−45.25), specificity 96.2% (95% CI: 93.9−97.9), positive predictive value 40.0% (95% CI: 21.1−61.3) and negative predictive value 93.6% (95% CI: 90.8−95.8) (Table 5). For the Tzanck test, its sensitivity compared with the M-PCR was 43.8% (95% CI: 37.5–50.3), specificity 88.9% (95% CI: 83.2−93.2), positive predictive value 84.8% (95% CI: 77.3–90.6) and negative predictive value 52.8% (95% CI: 46.8–58.7).

**Table 5:** Accuracy of syphilis serology and Tzanck test among patients attending an STI clinic in Manaus, Brazilian Amazon.

Assay	Tests characteristics (number of correct/total)			
	Sensitivity (95% CI)	Specificity (95% CI)	PPV (95% CI)	NPV (95% CI)
Tzanck test	43.8 (37.5–50.3) (106/242)	88.9 (83.2–93.2) (152/171)	84.8 (77.3–90.6) (106/125)	52.8 (46.8–58.7) (152/288)
Syphilis serology	27.8 (14.2–45.2) (10/36)	96.2 (93.9–97.9) (383/398)	40.0 (21.1–61.3) (10/25)	93.6 (90.8–95.8) (383/409)

CI: confidence interval; +: postive; −: negative. PPV: positive predictive value; NPV: negative predictive value.

## Discussion

This is the first study of GUD etiology in Brazil and it reports several relevant findings. Genital herpes, in particular HSV-2, was the leading cause of GUD among STI patients in Manaus, consistent with findings from several countries. [2], [20], [21], [22], [23] In studies from Africa, genital herpes accounted for more than three quarters of all cases of GUD. [24], [25] GUD due to HSV was detected more frequently in females than in males. Several previous studies have documented a higher seroprevalence of HSV-2 infection in women than in men. [26].

In most countries around the world syphilis ranks the second most frequent cause of GUD, with a worldwide prevalence in patients ranging from 2 to 25%. [2], [21], [22], [27] In the STIs clinic in Manaus, the prevalence was 8.3% as measured by PCR and 5.8% as determined by serology in a partially overlapping patient group (2.3%). No chancroid was identified among the participants with GUD, which is in agreement with other studies. [23], [28], [29] The highly sensitive molecular diagnostic method used in this study and considering that patients with chancroid are very likely to seek symptomatic relief, lead us to believe that the prevalence of chancroid could be negligible in Manaus.

In the past decade, other reports worldwide have observed a remarkable increase in HSV-2 and a decline in chancroid and other bacterial STIs. [1], [30] While the exact cause of this shift is not known, some of the factors that may explain this are improved bacterial STD management and improved HSV-2 diagnostic tools, [31] combined with changes in sexual behavior that could have promoted the transmission of genital herpes. [32].

HIV prevalence was higher than that in the general Brazilian population, in particular among men (3.5%) and in patients with GUD by HSV-2 (61.5%). In our study, the presence of clinical GUD was not associated with HIV infection. This result is of interest since a number of studies suggest that HSV-2 and syphilis are strongly associated with HIV acquisition despite the fact that the mechanism is not fully defined. [7] However, two recent studies also found no association between GUD and HIV seropositivity. [25], [33] A previous systematic review highlighted a clear publication bias in studies addressing HIV-STI interactions. [34].

In the multivariable model, genital herpes was associated with multiple and vesicular lesions that were recurrent and of a shorter duration. Given that herpes lesions are usually painful, patients may present for care earlier. Syphilis was associated with first episode and the lesion being unique. Several previous studies have shown the absence of strong clinical predictive factors for a definitive diagnosis of GUD. [25], [33], [35] However, findings from Malawi suggest that the use of unweighted algorithms containing basic clinical information may improve diagnostic accuracy of GUD. [1].

Overall, 32.5% of all ulcer specimens were found to be negative for all three etiological agents tested, which is consistent with reports from India and Malawi. [1], [20] Other studies in Africa have shown a higher proportion of ulcers where no pathogens were detected (51%–55%), [28], [36], [37] whereas a recent study in South Africa reported a lower estimate (18%). [38] Ulcers with negative PCR results may be due to: (1) alternative etiologies that were not tested, such as donovanosis or lymphogranuloma venereum: however, surveillance data from the STI clinic in Manaus did not report cases of such uncommon organisms during the study period. [39] Among 53 women seeking medical care for GUD in Fortaleza, Brazil, 45.3% GUD resulted from non-STD causes [40]; (2) an inadequate sample collection technique which is unlikely because only specimens that tested positive for actin [beta] gene, the DNA internal extraction control, were included in the analysis; (3) presence of PCR inhibitors which is unlikely given that all samples that were negative in M-PCR were positive for the human endogenous control; and (4) the pathogens had already been cleared by treatment since we did not exclude patients that had taken medication before the study. In many patients with ulcers of unknown cause, HSV-2 may have been the responsible pathogen. The Tzanck test was positive in 12.6% of patients with PCR-negativity which suggests that HSV may have been the etiologic pathogen. Some authors have found an association between having no pathogen detected and old ulcers for HSV-2. [41].

The introduction of the M-PCR was an improvement over the standard methods since there were more patients with GUD for whom a definitive etiology was found. In addition, M-PCR allowed for the identification of primary syphilis prior to the development of antibodies, which has important implications for early treatment. In the current study, discrepant results were obtained from 26 M-PCR TP positive patients who had a negative syphilis serology (VDRL and FTAbs). These results indicate that there might be low levels of viable or nonviable organisms that might have failed to elicit an immunologic response, which is common in GUD caused by TP, or may represent false positive results. The low syphilis reactivity rate (40%; 10 of 36) among M-PCR TP-positive ulcer cases could be explained by the window period in primary syphilis [42] or by previous treatment that might have resulted in immunologic nonresponse in these patients. The M-PCR did not detect *T. pallidum* in 15 specimens that were positive by syphilis serology. This may have been due to a false negative result by M-PCR, a treated syphilis or positive serology due to cross reactivity with other infections. Of these 15 specimens, 6 (40%) had a VDRL title >8. PCR assay has shown a correlation of 88% with RPR or VDRL serology without confirmation by treponemal antibody test [14] and of 95% when compared with a combination of serological tests. [43] The Tzanck failed to detect 47.2% of the specimens’ positive by M-PCR. Detection of HSV by M-PCR almost doubled detection by the Tzank test and might be due to detection of small amounts of herpes DNA present in ulcers which may be in various stages of disease. [44] Accurate HSV serologic tests are appropriate in asymptomatic cases, when viral and PCR assays are largely negatives, [45] but test only latent infection and may not detect recent seroconversion. [46].

The M-PCR procedure for GUD pathogens detection can be further improved if adapted to a probe-based real-time PCR assay, including a human housekeeping gene such as actin-beta as an internal control. Real-Time PCR has many advantages over traditional PCR including speedup, reduced risk of cross-contamination and broad dynamic range of target quantification, [47] which may be useful if it is necessary to monitor the amount of pathogen DNA during treatment.

The study had some limitations. Sexual behavior factors were not addressed in the study. Secondly, the estimate of etiology is derived from an STI clinic population and may not represent epidemiology of GUD in Manaus. Despite the fact that this clinic is the primary site for STI care in Manaus, the study could have been subject to selection bias since not all patients with GUD seek care. In a study among high-risk men and women in urban Peru, 62.6% of all symptomatic participants with GUD took some curative action about symptoms. [48].

In 2008, a recommendation was made during a World Health Organization expert meeting, to modify the GUD syndromic management guidelines to include anti-herpetic therapy as part of first-line GUD therapy, without a prevalence threshold. [49] Results from the “Partners in Prevention HSV/HIV transmission trial” showed that suppressive therapy did not decrease HIV transmission. [50] However, the decision was based on high HSV-2 prevalence among GUD patients in all settings, clinical benefits for those treated, and a potentially favourable cost-benefit profile. The addition of acyclovir was found to increase the number of ulcers correctly treated thereby reducing the cost per ulcer treated in certain scenarios. [51] So far, few countries have taken up this recommendation. [25], [49] Brazil is currently employing the 2003 WHO syndromic management that only includes antiviral treatment for HSV2 for anyone with typical symptoms/signs. [4].

In light of the etiologic findings in our study, STI treatment guidelines in Brazil may need to be revised and include anti-herpetic therapy in the syndromic management cocktail. Given that chancroid was not detected, the addition of treatment for *H. ducreyi* may not be necessary. However, local up-to-date surveillance would be essential to monitor any re-emergence of chancroid. The success of syndromic management of STIs is dependant upon education of patients to recognize the early symptoms of the infection and acyclovir becoming cheaply and widely available. Currently, there is a lack of consensus on how many studies and what prevalence threshold should guide the removal of antibiotics for low-prevalence ulcer pathogens. However, our findings should ideally be validated by conducting similar studies in multiple sites in Brazil.
